# Bioactivity of Argentinean Essential Oils Against Permethrin-Resistant Head Lice, *Pediculus humanus capitis*

**DOI:** 10.1673/031.010.14145

**Published:** 2010-10-22

**Authors:** Ariel C Toloza, Julio Zygadlo, Fernando Biurrun, Alicia Rotman, María I Picollo

**Affiliations:** ^1^ Centro de Investigaciones de Plagas e Insecticidas (CITEFA-CONICET), Juan Bautista de La Salle 4397, B1603ALO, Villa Martelli, Buenos Aires, Argentina; ^2^ Cátedra de Química Orgánica, Facultad de Ciencias Exactas, Físicas y Naturales, Universidad Nacional de Córdoba, IMBIV-CONICET, Córdoba, Argentina; ^3^ Laboratorio de diversidad vegetal y fitosociología, INTA EEA La Rioja. Argentina; ^4^ Cátedra de Botánica General. Universidad Nacional dejujuy. Argentina

**Keywords:** human head louse, Argentinean plants, biopesticides, fumigant activity

## Abstract

Infestation with the head louse, *Pediculus humanus capitis* De Geer (Phthiraptera: Pediculidae), is one of the most common parasitic infestation of humans worldwide. Traditionally, the main treatment for control of head lice is chemical control that is based in a wide variety of neurotoxic synthetic insecticides. The repeated overuse of these products has resulted in the selection of resistant populations of head lice. Thus, plant-derived insecticides, such as the essential oils seem to be good viable alternatives as some have low toxicity to mammals and are biodegradable. We determined the insecticidal activity of 25 essential oils belonging to several botanical families present in Argentina against permethrin-resistant head lice. Significant differences in fumigant activity against head lice were found among the essential oils from the native and exotic plant species. The most effective essential oils were *Cinnamomum porphyrium*, followed by *Aloysia citriodora* (chemotype 2) and *Myrcianthes pseudomato*, with KT_50_ values of 1.12, 3.02 and 4.09; respectively. The results indicate that these essential oils are effective and could be incorporated into pediculicide formulations to control head lice infestations once proper formulation and toxicological tests are performed.

## Introduction

Infestation with the louse, *Pediculus humanus capitis* De Geer (Phthiraptera: Pediculidae) is one of the most common parasitic infestation of humans worldwide (Burgess 2004). Children 3–12 years of age are the most affected group in both developed and developing countries. The main symptoms associated with infestation are constant itching, scalp irritation and social sanctioning. In rare cases, the itch-scratch cycle can lead to secondary infection with impetigo and pyoderma (Mumcuoglu et al. 2009). Transmission of head louse occurs mainly by direct host-to-host contact (Takano-Lee et al. 2005). Moreover, Falagas et al. (2008) reported that head louse infestation has been increasing worldwide due to the lack of effectiveness of pediculicides.

Traditionally, the main treatment to control head lice is chemicals including a wide variety of neurotoxic synthetic insecticides such as DDT, lindane, malathion, carbaryl, permethrin and δ-phenothrin (Burgess 2004). The repeated overuse of these products has resulted in the selection of resistant populations of head lice in several countries, including Argentina (Mumcuoglu et al. 1995; Picollo et al. 1998; 2000; Kasai et al. 2009). There is a strong consumer pressure against insecticide use that impacts health, the food supply, water and the environment (Isman 2008). Thus, there is an urgent need to find and develop new pediculicide substances. Plant essential oils and their constituent compounds such as the monoterpenoids seem to be good candidates because many are easily extractable, are biodegradable, and are very effective against a wide spectrum of insect pests (Isman 2000; Rahman and Talukder 2006), including head lice (Priestley et al. 2006; Rossini et al. 2008). In a previous study, we reported the fumigant and repellent activity of many native aromatic plants belonging to six botanical families against head lice (Toloza et al. 2006). In order to continue this work, the purpose of the current study was to assess the insecticidal activity of essential oils from native and cultivated aromatic plants from Argentina for their activity against permethrin-resistant head lice.

## Methods and Materials

### Insects

Head lice were collected from heads of 2,120 infested children 6–13 years old, using a fine toothed antilouse metallic comb. Lice were obtained from three elementary schools (HB, PR and E14) located in Buenos Aires, where a topical method indicated high resistance levels to permethrin (71.42, 35.37 and 33.33; respectively) (Toloza AC, unpublished). Briefly, this method consisted of the topical application of serial dilutions of permethrin in acetone. They were applied to individual lice with a 5-µl syringe, and each louse was treated with 0.1µl of the solution on the dorsal abdomen. Each concentration was replicated at least three times using 10 adults per replicate. Once collected, head lice were transported to our laboratory according to Picollo et al. (1998, 2000). The protocol for lice collection was approved by the ad hoc committee of the Centro de Investigaciones de Plagas e Insecticidas (CIPEIN, Buenos Aires, Argentina), and archived in our laboratory. After collection, head lice were maintained without feeding in an environmental chamber (Lab-line Instruments, www.lab-line.com) at 18 ± 0.5°C and 70–80% RH in darkness.

### Essential oils

Twenty five species of native and exotic plants in thirteen different families were collected in the spring of 2006 and 2007 from different regions of Argentina. Four of these plants, *Aloysia citriodora, Satureja parvifolia, Baccharis salicifolia* and *Chenopodium ambrosioides*, were the most effective. They were obtained from different environmental areas that allowed separating them into different chemotypes.

Voucher specimens were deposited at Jujuy Herbarium (Index herbarium code JUA) and INTA EEA La Rioja Herbarium (CHAM). The species are listed by family in Table 1 and the Appendix.

Dried leaves of each individual species were hydrodistilled in a Clevenger-like apparatus for 1 h. The oils obtained were dried over anhydrous sodium sulphate (Merck, www.syngentacropprotection.com) and stored in a refrigerator until analysis.

### Gas chromatography (GC)

Analyses of essential oils were performed in a Shimadzu GC-R1A (FID) gas-chromatograph (www.shimadzu.com), fitted with a 30 m × 0.25 mm (0.25 µm film thickness) fused silica capillary column coated with a phase 5% phenyl 95% dimethylpolysiloxane, non polar DB-5 column. The GC operating conditions were as follows: oven temperature programmed from 40 -230° C at 2° C/min, injector and detector temperatures 240° C. The carrier gas was nitrogen at a constant flow of 0.9 ml/min, and 0.3 µl of each material was injected into the Chromatograph. The constituents of the essential oils were identified on the basis of: (1) their GC retention index with reference to an homologous series of n-alkanes (C12 – C25); (2) by comparison of their retention times with those of pure authentic samples from Sigma and Fluka Companies; (3) peak enrichment on co-injection with authentic standards wherever possible; (4) by GC-MS library search (Adams and Nist); and (5) using visual inspection of the mass spectra from literature, for confirmation. Analyses were performed with a Perkin Elmer Q-700 (www.perkinelmer.com) equipped with a SE30 capillary column (30 m × 0.25 mm; coating thickness 0.25 µm film). The analytical conditions were: oven temperature from 40° C to 230° C at 2°C/min, the carrier gas was helium at a constant flow of 0.9 ml/min, the source was at 70 eV.

### Bioassay

The method of Toloza et al. (2006) was employed to evaluate the fumigant activity of the essential oils. Briefly, it consisted of an enclosed chamber (9 cm diameter Petri dish) that allowed creating a saturated microatmosphere as a result of the evaporation of the essential oil, thus exerting inactivation on lice. A drop of 60 µl of pure essential oil was deposited on a micro coverglass within the chamber. Control consisted of the same experimental unit without the addition of any substance. In each chamber 15 adult insects (males and females) were placed and exposed to the vapors of the essential oils. Lice were observed for evidence of knockdown every 5 min. for 60 min. The criterion for knockdown was when an insect remain on its back with no leg movements. Once used in a given assay, the insects were discarded. Three replicates were made for each tested essential oil. During each study, the assembled units were kept at 28 ± 1° C and 60 ± 5% RH.

### Statistical analysis

Probit analysis (Litchfield and Wilcoxon 1949) was used to estimate time in minutes to knockdown of 50% of exposed insects of each experimental unit (KT_50_), by using POLO Plus v2.0 (LeOra Software 2002). Insects exposed to test essential oils that did not show evidence of efficacy in 60 min, were considered to possess a KT_50_> 60 min and were not included in further statistical analysis. Samples for which the 95% fiducial limits did not overlap were considered to be significantly different.

## Results

Significant differences in fumigant activity against head lice were found among the essential oils from the native and exotic plant species (Table 1). Eighteen of the twenty five studied essential oils were native from Argentina (72.0%). It is important to note that 75% of the effective essential oils (KT_50_ <60) were from plants native to Argentina. On the basis of effectiveness, the essential oil from the native *Cinnamomum porphyrium* was the most effective (KT_50_= 1.12 min), followed by *A. citriodora* (chemotype 2) and *Myrcianthes pseudomato*, with KT_50_ values of 3.02 and 4.09; respectively. There were significant differences among all the studied chemotypes (Table 1). For example, *A. citriodora* (chemotype 2), *S. parvifolia* (chemotype 2) and *C. ambrosioides* (chemotype 1) showed pediculicidal action, with KT_50_ values of 3.02, 32.06 and 42.03 min; respectively. This is in contrast to the chemotypes of *A. citriodora* (chemotype 1), *S. parvifolia* (chemotype 1) and *C. ambrosioides* (chemotype 2) that possessed KT_50_ >60 min. Both chemotypes 1 and 2 of the essential oil *B. salicifolia* showed no action against head lice.

**Table 1.**
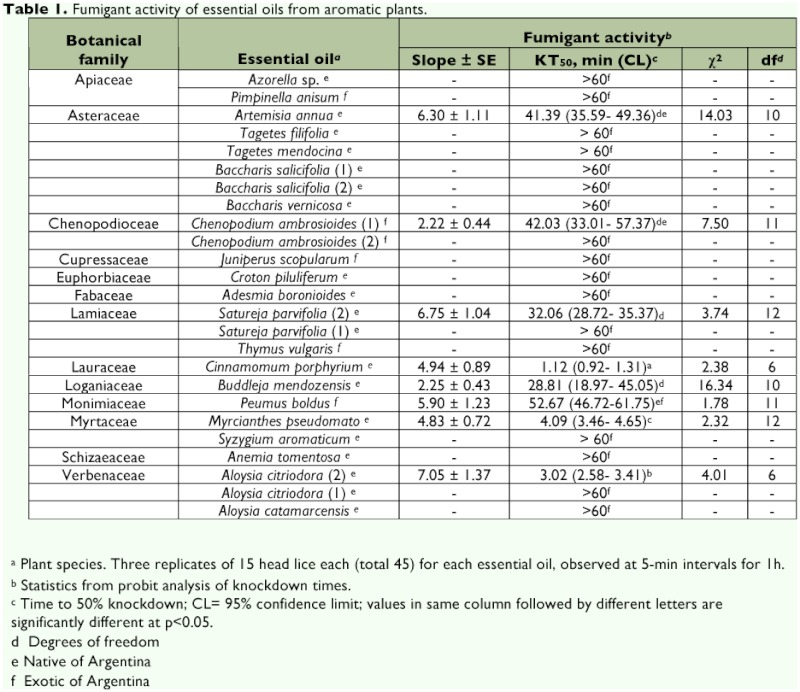
Fumigant activity of essential oils from aromatic plants.

## Discussion

The present study shows that essential oils from some Argentinean aromatic plants have fumigant activity against head lice. In comparison with our previous work (Toloza et al. 2006), new botanical families have been examined including Loganiaceae, Asteraceae, Monimiaceae, Apiaceae, Cupressaceae, Euphorbiaceae, Fabaceae and Schizaeaceae. This is the first study related to the pediculicidal efficacy of these botanical families. An interesting result was the fumigant activity of the three most effective essential oils from *C. porphyrium, A. citriodora* (chemotype 2) and *M. pseudomato*, which were 25.7-, 9.5-, and 7-fold more toxic than the oil of *Buddleja mendozensis*.. Yang et al. (2005) studied the fumigant activity of an essential oil from another plant of the same genera, *Cinnamomum zeylanicum*, which was also shown to be effective against head lice. However, a comparison with our results was not possible due to differences in the methodology.

Concerning the variation in chemical composition of the essential oils, it is important to note that the differences found in the fumigant efficacy of the oils *A. citriodora, S. parvifolia, C. ambrosioides*, are related to their chemical composition. For example, both chemotypes of *C. ambrosioides* had a very different chemical composition. Chemotype 1 possess as their main compounds *trans*carveol (42.4%), *trans*-pinocarvyl acetate (22.4%) and *cis*-carveol (10.6%), while chemotype 2 has ascaridol as its main compound (99.4%). However, other factors such as the interactions among the constituents could also affect the biological activity of the whole oil (Burgess 2004). Both chemotypes of *S. parvifolia* had similar chemical composition whose main compounds were piperitone and piperitenone oxide. However, chemotype 2 had a higher proportion of piperitone than chemotype 1 (46.0 and 41.9%; respectively) and a lower proportion of hydrocarbons and oxygencontaining sesquiterpenes (Dambolena et al. 2009). These differences were likely responsible for effectiveness of chemotype 2 against head lice.

There are several well documented studies showing that plant extracts could be used as medicinal products against a broad spectrum of ectoparasites of humans and house animals (Semmler et al. 2009; Burgess 2009). However, most of the mentioned products are derived from fixed oils rather than essential oils. For example, a product containing a neem seed extract was highly effective in *in-vivo* and *in-vitro* tests against head lice (Heukelbach et al. 2006; Abdel-Ghaffar and Semmler 2007). Recently, Burgess et al. (2010) showed in a clinical trial the superiority of a spray containing coconut, ylang ylang and anise oils over a permethrin lotion against head lice.

Our study indicates that certain essential oils from local plants of Argentina were highly effective in the vapor phase against head lice. However, *in vitro* efficacy tests of botanical extracts are only the first step of research and much work is needed before they could be used in a commercial product. The incorporation of excipients (alcohols, etc.) that increase the stability of essential oils is of a great concern since essential oils are highly volatile, and the effectiveness of the product could decay in hours if the formulation is incorrect. For instance, active ingredients that were effective *in vitro* could show low or no activity against lice when incorporated into a liquid formulation because certain adjuvants or excipients could affect the insecticidal activity once they are incorporated into a formulation. Once the vehicle base and the excipients are selected, a battery of tests for acute and chronic toxicity (e.g., burning sensation, skin irritation, etc) is needed. A final step should consider the *in vivo* efficacy of the product (i.e. in clinical trials).

The present work showed that basic research on essential oils toxicity against head lice should be considered only if the steps mentioned above are completed.
